# A Narrative Review of Stem Cell‐Derived Exosomes for Diabetic Nephropathy

**DOI:** 10.1155/sci/4021805

**Published:** 2026-07-14

**Authors:** Mo Li, Tianjing Sun, Allen Gao, Andrew Huang, Gagan Deep, Yuanyuan Zhang, Anyong Yu

**Affiliations:** ^1^ Department of Emergency, Affiliated Hospital of Zunyi Medical University, Zunyi, 563003, Guizhou, China, zmchospital.com.cn; ^2^ Department of Urology, University of California, Davis, Sacramento, California, USA, berkeley.edu; ^3^ Department of Ophthalmology and Visual Sciences, Washington University School of Medicine, St. Louis, Missouri, USA, wustl.edu; ^4^ Department of Internal Medicine-Gerontology and Geriatric Medicine, Wake Forest University School of Medicine, Winston-Salem, North Carolina, USA, wakehealth.edu; ^5^ Wake Forest Institute for Regenerative Medicine, Wake Forest University Health Sciences, Winston-Salem, North Carolina, USA, wfu.edu

**Keywords:** DN, exosomes, mesenchymal stem cells, pluripotent stem cells, stem cell therapy, therapeutic effect

## Abstract

Diabetic nephropathy (DN) remains a major cause of end‐stage kidney disease. Stem cell‐derived exosomes have emerged as a promising therapeutic strategy due to their ability to deliver bioactive molecules to damaged tissues. This narrative review, conducted in accordance with PRISMA guidelines, aims to evaluate the therapeutic potential of exosomes derived from various stem cell sources, including mesenchymal stem cells (MSCs), embryonic stem cells (ESCs), and induced pluripotent stem cells (iPSCs), in the context of DN. A comprehensive literature search was performed using relevant databases (e.g., PubMed, Web of Science, Scopus) to identify preclinical and clinical studies investigating the effects of stem cell‐derived exosomes on DN. The identified studies were assessed for quality and methodological rigor. Priority was given to high‐impact studies and those with robust experimental evidence. The selected literature was synthesized thematically to provide a coherent overview of the current state of research on exosome‐based therapies for DN, highlighting current findings, and future directions. Results from preclinical studies suggest that exosomes derived from different stem cell sources can exert reno‐protective effects, including reducing inflammation, fibrosis, and oxidative stress. However, comparisons between different exosome types indicate that MSCs‐derived exosomes (MSC‐Exos) may offer superior therapeutic benefits. While clinical trials are ongoing to evaluate the safety and efficacy of stem cell‐derived exosomes in DN patients, further research is needed to optimize exosome production, delivery, and therapeutic efficacy.

## 1. Introduction

### 1.1. Overview of Diabetic Nephropathy (DN)

DN is a common complication of diabetes as a major cause of end‐stage renal disease (ESRD) all around the world. The incidence rate of DN varies depending on various factors, such as age, duration of diabetes, and level of glycemic control. Approximately, the population of DN is comprised of 30% of people with type 1 diabetes and 40% of people with type 2 diabetes [1]. According to the study, DN is the main cause of ESRD, and ~10% of deaths in those are attributable to DN [2].

It is recommended that patients with diabetes undergo regular screenings and monitoring for DN to detect early signs of kidney damage and prevent or delay the progression of the disease, for example, the long‐term intensive control of glycemia, hypertension, and proteinuria [1]. Current treatment options for DN are limited and often have adverse side effects, which yet not slow or restrain the diabetes progress to end‐stage kidney failure [3]. Exosome therapy has emerged as a promising new approach for the treatment of DN. Exosomes are small extracellular vesicles (EVs) secreted by almost all cell types, which contain various bioactive molecules, such as proteins, lipids, and nucleic acids, that can modulate cellular functions and signaling pathways [4]. Specially, the exosomes derived from stem cells, both avoiding the inevitable limitations of stem cell therapy and inheriting similar therapeutic effects from their parental cell, have become a more promising alternative.

The varying cellular origin of exosomes contributes to their heterogeneity [5]. According to stem cells’ potency and source, the best examples of pluripotent stem cells (PSCs) including embryonic stem cells (ESCs) and induced PSCs (iPSCs) and adult multipotent stem cells including MSCs are delicate differences [6]. Understanding the differences in therapeutic potential of exosomes derived from different stem cell types is critical for the development of effective exosome‐based therapies for DN. This review aims to compare the therapeutic potential of exosomes isolated from MSCs and PSCs for DN and highlight the potential advantages and disadvantages of each approach. Preclinical studies have shown that MSC‐Exos have therapeutic potential for DN by reducing inflammation, oxidative stress, and fibrosis and promoting autophagic flux and M2 macrophage polarization in the diabetic patients, finally slowing the progression of DN [7, 8]. Recent studies have found that ESC‐derived exosomes (ESC‐Exos) exhibit a wide range of biological activities but are relatively understudied due to obvious ethical issues. Research on the application of PSC‐Exo in DN is still in its early stages, with direct literature being scarce. ESC‐EVs significantly promote the physiological repair and inhibit the pathological repair after acute kidney injury, enabling restoration of the structure and function of the damaged kidney [9]. iPSCs can clearly differentiate into pancreatic progenitor cells or insulin‐producing β‐cells in vitro [10]. In addition, we further analyzed and compared the advantages and disadvantages and therapeutic efficacy of stem cells and exosomes alone and their combination for DN, so as to identify a more optimized treatment for DN and guide conduct further research.

### 1.2. Pathological Changes and Molecular Mechanisms of DN

DN is a serious, chronic, and progressive disorder and has high morbidity and mortality [11]. Pathological structural changes occur in DN, including glomerular changes such as mesangial expansion, basement membrane thickening, and podocyte loss, as well as tubulointerstitial changes such as interstitial fibrosis and inflammation [12]. Other underlying mechanisms of DN including autophagy and oxidative stress, mitochondrial dysfunction, and abnormal crosstalk between renal cells play fat part in the progression of DN [13]. The importance of histopathological classification of DN and renal function should be monitored during follow‐up in the clinical setting.

DN is a leading microvascular complication of diabetes and a major cause of ESRD. In addition, the gold standard of diagnosing and dynamic assessment of DN is based on the pathological results of renal biopsy, which is an invasive and risky examination for patients. Furthermore, DN can quickly progress to ESRD without immediate therapy, so understanding the mechanism of the progression of DN is critically important for better treatment and prognosis. It was widely known that metabolic disorders, renal hemodynamics abnormalities [14], renin–angiotensin‐aldosterone system (RAAS) activation [15], mitochondrial oxidative stress, inflammation, etc., are currently recognized as the mechanism of DN.

**Figure 1:**
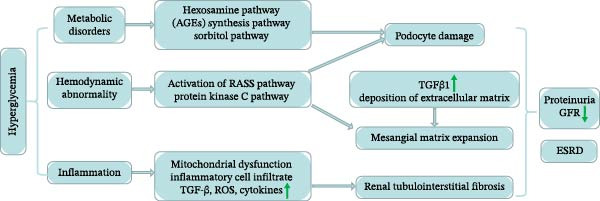
Pathological mechanism of DN. Abbreviations: AGEs, advanced glycation end products; ESRD, end state renal disease; GFR, glomerular filtration rate; RASS, renin–angiotensin–aldosterone system; ROS, reactive oxygen; TGF, transforming growth factor.

The molecular mechanism of DN including (ⅰ) metabolic abnormalities: high glucose (HG) induces mitochondrial oxidative stress in endothelial cells, which abnormally activates or inhibits downstream signaling pathways (such as hexosamine pathway, advanced glycation end products (AGEs) synthesis pathway, sorbitol pathway, protein kinase C pathway, etc.), and ultimately causes endothelial cell damage through inflammation and oxidative stress [16]. One of the recent understandings in the mechanism of DN is that glucose has been implicated in the disruption of endoplasmic reticulum (ER) response. Sustained metabolic abnormalities induce a chronic ER response, promoting the progression of DN [17]. (ⅱ) Hemodynamic abnormalities: The dysfunction of glomerular hemodynamics can be caused by the dysfunction of glucose metabolism and the excitation of RAAS. Disturbance of glucose metabolism leads to increased reactivity of a range of vasoactive factors that increase glomerular filtration pressure. High glomerular filtration increased the filtration of plasma albumin from the capillary walls. Glomerular hypertension can also affect the structure and function of glomerular intrinsic cells to varying degrees [18]. In addition, mechanical stress can exacerbate metabolic insults by stimulating excessive glucose uptake in cells. Just like there is a self‐sustaining cycle: hemodynamic stimulation on glomerular cells induces overexpression of the glucose transporter (GLUT‐1), followed by increased glucose uptake and activation of intracellular glucose metabolic pathways, resulting in excessive production of transforming growth factor (TGF‐β1). Conversely, TGF‐β1 maintains the overexpression of GLUT‐1, keeping the signaling sequence permanently active, as its ultimate effect, it increases the synthesis of extracellular matrix [15, 19]. (iii) inflammation and oxidative stress: Macrophages migrate and infiltrate the kidney tissue during early stages of DN, which promotes the release of TGF‐β, reactive oxygen species (ROS), vascular endothelial growth factor, and cytokines such as TNF‐α, subsequently promote the progression of DN [20, 21] (Figure 1).

The pathological process of DN is a complex and progressive one that involves a number of factors, including:

- Glomerular Basement Membrane (GBM) Thickening: In the early stages of DN, there are obvious changes in the podocyte–GBM interface such as thickening of the basement membrane that is one of the earliest manifestations (occur before microalbuminuria). No cellular proliferation or matrix deposition in the mesangium. The GBM’s structure may be significantly altered by an imbalance between ECM synthesis and degradation, nonenzymatic glycosylation, and change in some components (e.g., type IV collagen) across the GBM [22]. This thickening can impair the function of the glomeruli.
- Mesangial Expansion: Mesangial expansion is an important feature in the development of DN and is characterized by the abnormal proliferation of mesangial cells and matrix protein accumulation in the central region of the glomerulus. The mechanism of that is overexpression of TGF‐β1 promoting synthesis of fibronectin, collagen IV, laminin and glycated proteins trap ECM components. This expansion can also impair the function of the glomeruli. It is correlated with the macroalbuminuria and declining GFR.
- Glomerulosclerotic Lesions: Glomerulosclerotic lesions are the main pathological symbol of DN and an irreversible nephron destruction in the end‐stage of DN. Persistent and irreparable damage to endothelial cells and podocytes leads to the deposition of the ECM throughout the glomerulus. The above‐mentioned change can lead to the loss of functioning nephrons, which can eventually lead to kidney failure.
- Tubular and Interstitial Damage: The tubules and interstitium are the other parts of the kidneys that are responsible for filtering waste products from the blood. In DN, the tubule and interstitium can be damaged due to inflammation and fibrosis. This damage can lead to decreased kidney function.
- The pathological process of DN is a complex and progressive one that can lead to kidney failure. Early detection and treatment are essential for preventing kidney failure.

### 1.3. Current Treatment and Limitations of DN

The treatment of DN typically involves a combination of lifestyle changes, medications, and management of any underlying conditions. Here are some of the current treatments for DN: (1) *Blood sugar control*: Keeping blood sugar levels within a target range can help prevent or delay the chances of progression of DN [23]. This can be achieved through diet, exercise, and medications such as insulin or oral hypoglycemic agents. (2) *RAAS system inhibition*: high blood pressure is a common complication of DN and can further damage the kidneys. Medications such as ACE inhibitors or angiotensin receptor blockers (ARBs) can help lower blood pressure and ameliorate indirectly the effects of metabolic perturbations while rarely change the outcome of DN [24, 25]. (3) *Cholesterol management*: high cholesterol levels can also contribute to kidney damage in people with DN. Statins and other cholesterol‐lowering medications may be prescribed to manage cholesterol levels, which can protect the progression of DN through anti‐inflammatory, antioxidative, and antifibrotic effects [26]. (4) *Dietary changes*: A diet low in sodium and protein may be recommended to help reduce the workload on the kidneys and slow the progression of DN. (5) *Medications for anemia*: Clinical trials have been showed that anemia is frequently found in DN, occurs earlier and is more severe than in similar but non‐DN [27]. In addition, lower hemopoietin (EPO) concentrations were associated with rapid glomerular filtration rate (GFR) decline, especially in patients with iron deficiency [28]. Therefore, once anemia is present, medications such as erythropoietin‐stimulating agents (ESAs) may be prescribed to boost red blood cell production and improve renal function. (6) *Dialysis or kidney transplant*: In cases of severe kidney damage or ESRD, dialysis or kidney transplant may be necessary to replace the function of the kidneys [29, 30].

Despite currently available therapies have major advances in the treatment of DN, there remains a high residual risk of DN progressive progression [3]. It is important for people with DN to work closely with their healthcare providers to develop a personalized treatment plan that meets their individual needs and goals. Regular monitoring and follow‐up appointments are also important to track the progression of the disease and make any necessary adjustments to the treatment plan. However, this approach has been shown not to stop or reverse the disease [31]. Some studies discuss various challenges related to current treatments for DN, including the limited effectiveness of some medications, the difficulty in achieving optimal blood sugar and blood pressure control for individuals with multiple co‐morbidities and renal failure [32], and the heterogeneity of the disease. Additionally, some patients have a more limited administration of available medicine [33]. These challenges highlight the urgent need for ongoing research to develop more effective treatments for DN and to better understand the underlying mechanisms of the disease.

## 2. The Potential of Stem Cell‐Derived Exosomes as a Novel Therapeutic Approach

Stem cells can self‐renew and develop into a variety of functional cells, which are considered potential therapeutic agents for DN due to their beneficial effects mainly through paracrine mechanisms [34, 35]. Exosomes derived from various sources, including mesenchymal stem cells (MSCs), have been considered as potential therapeutic agents for DN in preclinical studies, but clinical trials on DN have not been conducted.

There are several studies showing that exosome therapy has been investigated as an emerging and potential treatment in various disease models, including osteoporosis, atherosclerosis, hepatocellular carcinoma, and kidney injury [36–39]. Increasing preclinical studies have demonstrated that exosomes can have therapeutic effects through various mechanisms, such as promoting tissue repair and regeneration [40], reducing inflammation [41], and modulating immune responses [42].

The mechanism of exosome therapy for DN is still being investigated. Here are some potential mechanisms of exosome therapy for DN: (1) *Modulation of inflammatory and immunological effects*: Exosomes can contain anti‐inflammatory molecules such as miRNAs, cytokines, and chemokines, which can reduce inflammation in the kidneys. Chronic inflammation is a key contributor to the development and progression of DN, and reducing inflammation can help to slow or halt the disease process [43]. Macrophages, as the predominant innate immune cells of DN, are commonly observed in the glomeruli and interstitium in experimental DN models and clinical pathological biopsy. AGEs can stimulate the expression of intercellular adhesion molecule (ICAM‐1) and Monocytechemoattractantprotein‐1 (MCP‐1) in renal tubular cells in the diabetic environment, thereby promoting the recruitment of macrophages and ultimately aggravating renal fibrosis [44]. Macrophage therapy may have therapeutic effects on diabetic kidney disease (DKD) by regulating the inflammatory response in the kidneys caused by the diabetic environment [45]. Preclinical study shows that after HG pre‐treatment, EVs miR‐21‐5 p derived from macrophages increased the levels of inflammasome nucleotide‐binding oligomerization domain‐like receptor protein 3 (NLRP3) and Interleukin‐1β (IL‐1β) in DN model mice and in vitro, thereby inducing pyroptosis of podocytes by targeting A20, which plays an important role in inflammation‐mediated DN [44, 46]; (2) *induction of autophagy：*Exosome induced autophagy can attenuate the renal injury in the models of DN by suppressing the mammalian target of rapamycin (mTOR) pathway [47]; (3) *inhibiting apoptosis*: Overexpressed miR‐16‐5p in exosomes may have the potential to prevent kidney injury from diabetes by suppressing VEGFA expression and preventing apoptosis of podocytes induced by HG [48, 49]; and (4) *promoting normal vascular regeneration*: study provides evidence that exosome miR‐30a‐5p suppresses abnormal angiogenesis of renal endothelial cells by modulating the Notch1/VEGF signaling pathway [50].

Moreover, further research is needed to optimize the production and delivery of exosomes and to better understand their mechanisms of action in DN. In short, the mechanism of exosome therapy for DN is complex and multifactorial, and it is likely that multiple pathways are involved in its therapeutic effects.

### 2.1. Mechanisms of Action of MSC‐Exos in DN

MSC‐Exos have been shown to have therapeutic effects in DN. Here are some of the proposed mechanisms of action of MSC‐Exos in DN: (1) *modulation of immune inflammatory responses*: Increasing evidence indicates that the ongoing injury of DN is contributed to the chronic inflammatory stimulation [51]. For example, Macrophages, the important part of regulating immune inflammatory response, migrate and infiltrate the kidney tissue during early DN [20], which secrete and releases the TGF‐β, VEGF, and cytokines such as TNF‐αand IL‐1, subsequently accelerating the progression of DN [21]. MSC‐Exos contain anti‐inflammatory molecules, such as microRNAs and cytokines, which can reduce inflammation in the kidneys. Inflammation is a major contributor to the development and progression of DN [52]; (2) *Anti-fibrotic effects*: MSC‐Exos can secrete some factor‐induced fibrosis, such as downregulating snail and collagen‐1 expression and up‐regulating Serpina1α and FAS ligand et al., which can prevent the development and progression of fibrosis in the kidneys. Also, the anti‐fibrotic impact of MSC‐Exos was related to the downregulation of various pro‐fibrotic genes in kidney tissues [53]; (3) *regeneration of normal blood vessel*: It is reported that microangiopathy in patients with diabetes leads to thickening of the glomerular capillary basement membrane and dilation of the mesangial matrix [54]. Therefore, it is important to ensure the normal blood vessel formation in the kidney for maintaining renal function. It is shown that exosomes from urine‐derived stem cells carrying potential factors (high levels of VEGF and angiogenin) may promote normal angiogenesis and podocytes survival and be beneficial for renal protection in diabetes [49]. (4) *Anti-apoptosis and inducted autophagy*: MSC‐Exos contain factors that can directly targeted effect the autophagy factor, such as Smad1/mTOR and hyperglycemia‐induced‐tumor necrosis factor receptor‐associated factors (TRAF6), and subsequently induct autophagy and prevent apoptosis (cell death) of kidney cells, which can promote cell survival and regeneration [55].

Overall, MSC‐Exos have the potential to ameliorate and prevent the pathological changes associated with DN through the mechanism described above and subsequently induce reno‐protective effects. However, further research is needed to fully understand their mechanisms of action and optimize their therapeutic potential.

### 2.2. Exosome Therapy in Clinical Trial and Preclinical Studies

Exosome therapy has shown promise in preclinical studies for the treatment of DN. Here are some details on these studies. For example, a preclinical study by Li et al. [56]. demonstrated that exosomes derived from MSCs can reduce renal fibrosis,promote the polarization of macrophages (such as promoting transformation of the macrophage cell from M1 type to the M2 type), and reduce inflammation in a mouse model of DN. Moreover, Su et al. [57] have shown that exosomes secreted by MSCs pre‐treated with a diabetic environment (Exo‐pre) have a more pronounced protective effect against DN by regulating the macrophage polarization. Exo‐pre administration exhibited a superior effect on DN by remodeling the macrophage balance by shuttling miR‐486‐5p, which targets PIK3R1.

Although, the exosome is employed as a promising and non‐invasive biomarker for diagnosis and follow‐up monitoring of DN. Little clinical trials have investigated the safety and efficacy of exosome therapy for the treatment of DN. One study found that miRNAs (miR‐1246, miR‐642a–3p, let‐7c‐5p, miR‐1255b‐5p, let‐7i–3p, miR‐5010–5p, and miR‐150–3p) that uniquely up‐regulated in DN patients compared to healthy volunteers and miR‐4449 that was highly expressed in DN patients compared to patients without DN. These miRNAs are significantly correlated with the degree of albuminuria and likely involved in MAPK signaling, integrin function in angiogenesis, and regulation of the AP‐1 transcription factor [58].

Although exosomes have a beneficial effect in the preclinical research of DN, there are few related clinical studies. It is critical that expanding the scope of exosomes in clinical studies provides more secure and rigorous data for clinical treatment.

### 2.3. Advantages and Limitations of Exosome Therapy

Exosomes are broadly categorized into natural exosomes and engineered exosomes depending on whether they have undergone artificial modification. The biogenesis of exosomes is a complex multistage process. The most common method of exosome formation, that is, an endosomal route including endosomal invagination, the development of early and late sorting endosomes, and ultimately the formation of multivesicular bodies (MVBs) containing intraluminal vesicles (ILVs). The early endosomes are produced by cell membrane invagination, during which the bioactive substances begin to accumulate within the early sorting endosomes. The late sorting endosomes then form MVBs after a second indentation. Finally, the MVBs fuse with the cell membrane, releasing the carried exosomes to the outside. As reported in the literature, classic exosomes contain numerous molecules, including proteins, glycoconjugates, lipids, nucleic acids, metabolites, and other bioactive substances. At present, the available traditional separation and production methods of exosomes are density‐ and size‐based techniques, including ultracentrifugation (UC), asymmetric flow field‐flow fractionation, size‐exclusion chromatography, ultrafiltration (UF), polymer‐based precipitation, etc. There are some shortcomings in the separation of exosomes, including low purity, contamination, coexistence of immunoglobulins, vesicle destruction, and heterogeneity, which affect the biological function of exosomes. New isolation strategies for exosomes show great progress in harvesting high‐quality EVs including microfluidics, asymmetric flow field‐flow fractionation (AF4), ion exchange, and combined multi‐step methods [59, 60]. Among the above new methods, microfluidics was used for exosome separation based on viscoelasticity, with a recovery rate exceeding 80% and a purity of over 90%. AF4 is an emerging technology based on EV that is the most commonly used method for separating EV subgroups. Iron exchange is an EV separation technique based on charge, using negatively charged EV membrane components (whose charge is determined by zeta potential [61]) and anion exchangers with positively charged functional groups to interact with cations. Compared with UC, this method only requires a shorter separation time (less than 3 h for 1 L of cell culture supernatant), and it can improve EV purity by removing viral contaminants and other proteins from the isolates. The combination of multiple methods is usually the best choice for EV separation as the sources of EV are diverse and their properties are complex and heterogeneous, requiring complex separation methods.

Exosome therapy is a promising approach for the treatment of DN, but like any therapeutic approach, it has both advantages and disadvantages. Here are some potential advantages and disadvantages of exosome therapy [62] (Table 1).

**Table 1:** Advantages and limitations of exosome therapy.

Advantages	Limitations
• Non‐immunogenic • High specificity • Safety (MSC‐Exos) and minimal side effects • Multi‐function • Less invasive Easy in storage and transportation	• Limited scalability • Complex formulation • Cost • Unknown long‐term effects (iPSC/ESC‐Exos)

Abbreviations: ESC‐Exos, embryonic stem cells derived exosomes; iPSC‐Exos, induced pluripotent stem cells derived exosomes; MSC‐Exos, mesenchymal stem cells derived exosomes.

Advantages include (1) As exosomes are natural vesicles that are produced by patient’s own cells and other compatible and suitable donors’, it is not expected to have significant adverse effects, such as the rejection response, on the body. In addition, the exosome therapy has reduced risk of tumor formation compared with the MSC. This makes exosome therapy a potentially safe treatment option for DN [63, 64]; (2) Exosomes can be engineered to target specific cells or tissues, which can increase the specificity and effectiveness of the therapy [4, 65]. (3) Depending on the cell from which they originate, exosomes carry complex molecular cargos such as proteins, lipids, RNA, non‐coding RNA, microRNA, and small RNA, which are carefully controlled by the parent cell allowing information relating to specific cellular functions. They activate the intracellular signaling pathways of the target cells, thereby influencing multi‐functions of the target cells such as immune regulation, anti‐inflammation, anti‐fibrosis, inhibition of oxidative stress, and enhancement of angiogenesis [4]. (4) Exosome can be purified and isolated from different source, including MSC or other iPSC, without the need for the sophisticated gene edit. Thus, exosome treatment can be stored and transported more easily than living cells [66].

Difficulties in production and formulation of exosomes: (1) The production of exosomes can be difficult and time‐consuming, which may limit the scalability of the therapy [67]; (2) The formulation of exosome‐based therapies can be complex, and the availability of exosomes for clinical use is limited, which may make it difficult to manufacture and standardize the therapy [68, 69]; (3) As exosome therapy is a relatively new approach, the long‐term effects of the therapy are not yet fully understood. Further research is needed to determine the optimal dose, delivery method, and therapeutic potential of exosome [70]; and (4), Exosome therapy and the manufacturing processes might be expensive [71, 72].

Thus, exosome therapy has many potential advantages for the treatment of DN, but there are also some potential disadvantages that need to be considered. Further research is needed to fully understand the risks and benefits of exosome therapy and to optimize its use as a therapeutic approach for DN.

### 2.4. Therapeutic Effect of Exosomes Isolated From Different MSCs

The prevalent types of MSCs are commonly derived from bone marrow, adipose tissue, and perinatal tissues (human umbilical cord, umbilical blood, placenta, etc.). Exosomes isolated from different cell types can differ in their origin tissue, composition, size, and biological functions. This is because exosomes are formed by budding from the plasma membrane of cells, and their content reflects the molecular and cellular characteristics of their parent cells.

Heterogeneity among exosome composition and function can affect their therapeutic potential and may have implications for their use in different disease contexts of DN. In general, while all MSC‐Exos share core therapeutic functions such as anti‐inflammation and tissue protection, significant differences exist among them, largely attributable to their tissue of origin. Bone marrow‐derived MSC exosomes (BMSC‐Exos), derived from a highly immunomodulatory and osteogenic niche, have been most extensively characterized for their broad‐spectrum cytoprotective effects, particularly through the modulation of fundamental processes like autophagy via the mTOR pathway. In contrast, adipose tissue‐derived MSC exosomes (ADMSC‐Exos), originating from metabolically active adipose tissue, appear to have a pronounced efficacy in protecting podocytes and modulating metabolic pathways relevant to diabetes, such as delivering miR‐486 to suppress the Smad1/mTOR axis. Meanwhile, umbilical cord‐derived MSC exosomes (UCMSC‐Exos), possessing a more primitive and immune‐privileged nature, demonstrate superior potency in mitigating specific inflammatory cascades, exemplified by their delivery of miR‐22–3p to inhibit the NLRP3 inflammasome. These functional predispositions suggest that the choice of the MSC source could be tailored to specific pathological aspects of DN.

Exosomes derived from different types of MSCs exhibit variability in therapeutic efficacy and mechanisms, which is described in detail in the following text (Table 2). While exosomes derived from BMSC‐Exos are the most commonly studied. BMSC‐Exos can interact with many types of cells and are not easily inactivated. They have the advantages of a high number of possible passages and stable biological performance [78]. BMSC‐Exos exert modulating autophagy, antifibrosis, anti‐inflammatory, and immunomodulatory effects through miRNA they carry and the regulation of signaling pathway. A study found that BMSC‐Exos could induce autophagy via the mTOR signaling pathway, significantly increasing the expression of Beclin‐1 in renal tissue, subsequently repairing renal function [47]. A study found that stem cell‐derived EVs miRNA significantly inhibit fibrosis and prevent its fibrosis during the process of diabetes‐induced chronic kidney injury [53]. Furthermore, the depletion of miR‐30e–5 p derived from BMSCs‐Exos promoted pyroptosis in HK‐2 cells induced by HG through inhibiting ELAVL1 [79].

**Table 2:** Therapeutic effect of different MSC‐Exos on DN.

Model	Administration (duration time)	Therapeutic effect	Mechanism	References
Ms	Tail vein (12 weeks)	Induction the podocyte autophagy and inhibiting apoptosis	Upregulate miR‐486 and inhibit Smad1/mTOR signal pathway	[8]
Rat	Tail vein (4 weeks)	Improve renal autophagy and fibrotic	Decrease mTOR and fibrotic marker expression	[47]
Cell culture	Co‐culture	MSC‐Exos protect GMCs	Via transferring of miR‐222 to targetedly downregulate STAT5 and reduce of TGF‐β and promote production of collagen within GMCs	[73]
Rat	Tail vein (3 weeks)	Suppress mesangial hyperplasia and kidney fibrosis	miR125a carried by Exos directly bound to HDAC1, and HDAC1 further regulated ET‐1 axis	[74]
Rat	Tail vein (12 weeks)	Alleviate podocyte apoptosis and enhance it proliferation	miR‐16–5p secreted by Exos inhibiting the expression of VEGFA in podocyte	[48]
Cell culture	To‐culture	Induce autophagy and inhibit apoptosis	Exosomal miR‐125b induced the Akt expression to inhibit apoptosis and downregulate the TRAF6 to inhibit the autophagy.	[55]
Ms	Tail vein (6 weeks)	Decreasing the apoptosis and inhibiting epithelial‐to‐mesenchymal (EMT) transition	Through exosomal miR‐424‐5p targetedly inhibiting YAP1 activation	[7]
Rat	Tail vein (2 weeks)	Decrease inflammation and fibrosis	Reduce the proinflammatory cytokines and pro‐fibrotic factor (TGF‐β).	[75]
Rat	Tail vein (2 weeks)	Attenuated inflammatory cell infiltration into the kidney tissue	Exosomal miR‐146a‐5p inhibiting the TRAF6/STAT1 to promote M2 macrophage polarization, result in reducing the local and systemic inflammatory cytokine levels.	[76]
Cell culture	Co‐culture	Exosomes secreted from high‐glucose‐treated glomerular endothelial cell plays a cross talk role to active EMT and fibrotic changes of mesangial cell and podocyte in DN.	Product α‐SMA expression and EM protein overproduction through the TGF‐β1/Smad3	[77]
Rat	Tail vein (12 weeks)	Inhibiting podocyte apoptosis and promoting vascular regeneration	Suppress the Caspase‐3 overexpression, USCs‐Exo contained the potential factors, including growth factor, TGF‐β1, angiogenin, and bone morphogenetic protein‐7, which may be related with vascular regeneration and cell survival.	[49]

Abbreviations: Akt, protein kinase; EMT, epithelial–mesenchymal transition; ET, endothelin; GMCs, glomerular mesangial cell; HDAC, histone deacetylase; Ms, mouse; mTOR, mammalian target of rapamycin; SMA, smooth muscle actin; STAT, signal transducers and activators of transcription; TRAF, tumor necrosis factor receptor‐associated factors; USC‐Exos, urine‐derived stem cells exosomes; YAP1, yes‐associated protein 1.

ADMSC‐Exos are abundant, easily purified, and associated with fewer ethical concerns compared to other MSC sources. ADMSC‐Exos can protect podocytes and renal tubular epithelial cells through various pathways by relieving inflammatory responses and subsequently ameliorate renal function. Podocyte damage plays a key role in the pathogenesis of DN. For example, ADMSC‐Exos vividly alleviated DN symptom by enhancing upregulation of miR‐486, which led to the suppression of the Smad1/mTOR signaling pathway in podocyte [8]. For instance, ADMSC‐Exos can transfer miR‐26a–5p into podocytes targeting TLR4, inactivating the NF‐κB pathway and downregulating vascular endothelial growth factor A (VEGFA), thereby enhancing cell viability and inhibiting apoptosis [80].

UCMSC‐Exos demonstrate lower adipogenic potential, low immunogenicity, and enhanced osteogenic capacity compared to other MSC types [81]. The inflammatory effect of UCMSC‐Exos cannot be ignored. Researcher found that under HG and DN conditions, miR22‐3p hUCMSC‐Exos can prevent podocytes from apoptosis and reduce the release of inflammatory factors (IL‐6, IL‐1β, IL‐18, and TNF‐α) caused by HG by inhibiting the activation of NLRP3 signaling [82].

Despite these differences, MSC‐Exos from all sources share fundamental mechanistic commonalities. They primarily function as intercellular signaling vehicles by delivering functional miRNAs to target cells, ultimately converging on the inhibition of key pathological processes in DN, including inflammation, apoptosis, and fibrosis. The heterogeneity in their cargo, rather than being a drawback, highlights a complementary relationship. It suggests the potential for a precision medicine approach, where the MSC‐Exos source could be selected based on the dominant pathological phenotype of an individual’s DN. Furthermore, the exploration of combination therapies using exosomes from different sources to simultaneously target multiple pathways represents a promising future direction. Ultimately, despite their distinct biological fingerprints, all MSC‐Exos face shared challenges in clinical translation, such as standardization and scalable manufacturing, which need to be addressed to harness their full therapeutic potential.

In conclusion, the differences and commonalities among exosomes from different MSCs have significant implications for precise clinical transition and treatment.

## 3. PSC‐Derived Exosomes in DN

IPSC are generated by reprograming adult cells and can differentiate into various lineages, even disease‐specific iPSCs (such as those for diabetes) [83]. However, present studies on iPSC‐Exos have primarily focused on extrarenal complications of diabetes, such as skin wounds and postoperative cognitive dysfunction, where they promote wound healing through immunomodulation, pro‐angiogenic effects [84] and demonstrated neuronal loss and reduced neurogenesis in diabetic mice model [85] and acute kidney injury [9]. And, the research on PSC in the field of DN is rather rare in present. For example, one study found that EVs derived from iPSCs could reduce RASS activation and fibrogenesis in an in vitro model of mesangial cell fibrosis, suggesting their potential as a therapeutic strategy for mitigating glomerulosclerosis [86]. Another study have shown that ESCs not only exhibit the highest potential to differentiate into insulin‐secreting cells, but can also be induced to form renal lineages using a defined set of growth factors or inducers [87]. More research on PSC should be allocated in the DN field in future. However, there still are several regenerative capacity differences between exosomes derived from ESCs/iPSCs and those from MSCs (see Table 3).

**Table 3:** Comparison of regenerative capacity between MSC‐Exos and iPSC/ESC‐Exos.

Regenerative capacity	MSC‐Exos	iPSC/ESC‐Exos
Regeneration potential^a^	Strong	Weak
Exosome isolation	Easy	Unwanted differentiation or tumorigenesis
Safety	Yes	Ethical and safety concerns
Levels of heterogeneity	Low	High, due to a wider range of differentiation capacity
Potential for inducing pluripotency in recipient cells	Low	High, induce the differentiation of somatic cells into iPSCs
Potential for cancer therapy	Low	High, due to carry tumor‐suppressing miRNA
Self‐renewal capacity after extended passaging	Low	High

^a^Regeneration potential: delivering bioactive molecules, i.e., growth factors, cytokines, and miRNAs, to the renal cells, and by modulating the immune response and antifibrosis.

In addition, one research study on the combination of PSC and MSC in DN has overcome the weaknesses of both, such as ethical and loss of self‐renewal capacity after extended passaging concerns. For example, one study shows that PSC‐MSC‐derived exosomes show the protective effect against the key steps of CKD, renal fibrosis, by the SIRT6/*β*‐catenin signaling pathway [88].

In recent studies, stem cell‐derived exosomes have emerged as a leading candidate for DN treatment in both preclinical and clinical research due to their distinctive advantages. These include the ability to target specific cells, the potential to slow CKD progression, minimal clinical ethical concerns, low immunogenicity, reduced risk of tumorigenesis, and long‐term tissue repair capabilities. Further in‐depth research is essential to explore the broader potential of stem cell‐derived exosomes and even their combination in DN therapy.

## 4. Exosomes With Combined With Other Therapies

### 4.1. Comparison of Exosome Therapy With Stem Cell Therapy in DN

Both exosome therapy and stem cell therapy are promising approaches for the treatment of various diseases, including DN. Compared to stem cell therapy, exosome therapy has potential advantages in terms of lower immunogenicity, simpler formulation, higher specificity, and potentially better safety profile. However, stem cell therapy has advantages in terms of greater regenerative potential, longer‐lasting effects, multifunctionality, and more established evidence for its safety and efficacy. Further research is needed to determine the optimal therapeutic approach for DN, including the use of exosome therapy, stem cell therapy, or a combination of both. Overall, exosome therapy is a promising therapeutic approach that may offer a new avenue for the treatment of DN (Table 4).

**Table 4:** Comparison of stem cell therapy and exosome therapy in DN.

Difference	Stem cell therapy	Exosome therapy
Advantages	‐ Well studied ‐ Renal cell differentiation potential ‐ Self‐renewal ‐ Longer‐lasting impact	‐ Less invasive ‐ Fewer safety concerns ‐ Off‐the‐shelf medicine ‐ No need for genetic manipulation or expansion ‐ Storage and transportation of exosome easily
Disadvantages	‐ Time‐consuming, ‐ Expensive ‐ Safety concerns in vivo ‐ Ethical concerns for iPSCs/ESCs ‐ Risk of immune rejection ‐ Low survival rate and off‐target effects	‐ Shorter half‐life ‐ Require multiple doses or sustained release formulations ‐ Limitations in severe tissue injury ‐ Safety concerns for ESCs/iPSCs‐exosomes

Abbreviations: ESCs, embryonic stem cells; iPSCs, induced pluripotent stem cells.

Here are several potential advantages of exosome therapy and stem cell ctively:

*Advantages of exosome therapy are* (1), Lower risk of immunogenicity: Exosomes are derived from cells and are not expected to have significant immunogenicity, which reduces the risk of rejection or other immune‐related complications [89]; (2), Simpler formulation: Exosome‐based therapies are generally easier to manufacture and standardize than stem cell‐based therapies, which can reduce the complexity and cost of the therapy; (3), Higher specificity: Exosomes can be engineered to target specific cells or tissues, which can increase the specificity and effectiveness of the therapy [90]; (4), Safer: Due to non‐cellular nature and controllable biological activity as characteristics of natural vesicles, it may have a better safety profile than stem cell therapy, which can have the potential risk of tumorigenesis or other adverse events [91].

*Advantages of stem cell therapy include* Greater regenerative potential: (1) Stem cells have the ability to differentiate into various types of cells and can potentially regenerate damaged tissues, which may make stem cell therapy more effective in promoting tissue repair and regeneration [92]; (2) Longer‐lasting effects: Stem cells can potentially continue to differentiate and regenerate tissues over a longer period of time, which may result in longer‐lasting therapeutic effects; (3) Multi‐functional: Stem cells can secrete a variety of therapeutic molecules, similar to exosomes, which can have multiple therapeutic effects on the body; and (4) More established: stem cell therapy has been used in clinical practice for a longer period of time and has been more extensively studied, which may provide more established evidence for its safety and efficacy [93, 94].

Briefly, both exosome therapy and stem cell therapy have potential advantages and disadvantages for the treatment of DN. The choice of therapy may depend on various factors, including the severity of the disease, the specific goals of therapy, and the availability and accessibility of therapies. Further research is needed to compare the safety and efficacy of exosome therapy and stem cell therapy in clinical trials and to determine the optimal therapeutic approach for DN.

### 4.2. Combination Therapy

The combination of stem cells and exosomes for the therapy of DN is an emerging area. While each approach has its own advantages and limitations, combining them may enhance their therapeutic effects and overcome their limitations. For example：

#### 4.2.1. Synergistic Effects

Stem cells and exosomes have complementary mechanisms of action and can work synergistically to enhance tissue repair and regeneration. The implanted MSCs can secrete a variety of beneficial factors (including endogenous exosomes, growth factors, and cytokines) at the injury site for a long time and continuously, providing a dynamic and adjustable treatment. The engineered exosomes can precisely deliver cargo biomolecules to target cells (such as podocytes and mesangial cells) by transcytosis through the endothelial layers [95]. In combination therapy, exosomes can quickly respond to acute injury signals and transmit crucial anti‐inflammatory and anti‐apoptotic instructions, while stem cells provide continuous support.

#### 4.2.2. Reduced Side Effects

While stem cell therapy has shown promising results for the treatment of DN, it also has some limitations, such as the risk of tumor formation and immune rejection. Exosomes, on the other hand, have low immunogenicity and can be derived from various cell types, including stem cells, without the risk of tumorigenesis. By using exosomes derived from stem cells, researchers can potentially overcome some of the limitations of stem cell therapy and reduce the risk of side effects [96].

#### 4.2.3. Enhance the Targeted Homing and Survival of Stem Cells

The transplanted MSCs showed limited aggregation in the kidneys and had a short retention time. Fluorescence tracking revealed that the transplanted MSCs had almost no renal homing function due to the first‐pass effect [97]. Administering exosomes either separately or simultaneously can alleviate the local inflammation and ischemic conditions in the kidneys, creating a more favorable environment for the subsequent infusion of MSCs and facilitating their survival and functional performance.

#### 4.2.4. Construct an “Enhancement‐Targeting” Delivery System Based on Engineered Exosomes.

That is, by applying engineering techniques to modify exosomes, they can not only directly treat renal cell but also act as delivery carriers to precisely deliver factors that promote survival and homing to the transplanted MSCs or target cells in the kidneys, forming a positive cycle [98].

### 4.3. Engineered Exosome for DN

To overcome the limitation of natural exosomes, such as non‐specific targeting and short retention time, researchers are developing engineering exosomes to enhance their therapeutic potential of DN. Despite numerous methods for obtaining engineering exosomes, exosome engineering strategies can be broadly categorized based on the stage of intervention: parent cell engineering (endogenous modification) and isolated exosome engineering (exogenous modification).

#### 4.3.1. Parent Cell Engineering (Endogenous Modification)

This approach genetically or epigenetically modifies the parent cells (e.g., MSCs) to produce exosomes with predefined characteristics. The engineered exosomes were then naturally secreted and harvested.

##### 4.3.1.1. Genetic Engineering

Expression vectors encoding targeting ligands (e.g., peptides and antibody fragments) or therapeutic proteins (e.g., Lamp2b and CD63) are fused with genes of exosomal membrane proteins and transfected into parent cells. The subsequent exosomes display these molecules on their surfaces. For instance, exosomes from ADSCs engineered to overexpress the long non‐coding RNA HOXB3OS were shown to ameliorate podocyte injury and DKD progression by suppress Ythdc2‐mediated SIRT1 mRNA degradation and counteracting SIRT1 downregulation under HG conditions [99]. While promising, the application of genetic engineering exosomes in DN therapy requires further exploration beyond its current prominence in oncology research.

##### 4.3.1.2. Preconditioning

These non‐genetic procedures rely on modulating the parental cell’s microenvironment to alter exosome cargo. Stimuli such as hypoxia, immunogenic cytokines, chemical agents, or physical stimuli can enhance the therapeutic potency of the secreted exosomes. For instance, a study found that preconditioned MSCs in a diabetic milieu release exosomes with heightened protective efficacy against DN, partly by modulating macrophage polarization [57].

#### 4.3.2. Isolated Exosome Engineering (Exogenous Modification)

This strategy directly modifies the purified exosomes after isolation.

##### 4.3.2.1. Cargo Loading

Therapeutic agents (drugs and nucleic acids) are actively loaded into the exosomal lumen. Electroporation is a common, controllable, and highly efficient technique that uses electrical pulses to create transient pores in the exosomal membrane for cargo entry. It allows for adjusting various parameters, such as voltage, current, and pulse, to achieve the desired quantity and quality of exosomes. A study demonstrated that exosomes from hUCMSCs (hUCMSCs‐Exo) loaded with Ex‐4 via electroporation promoted the induction of CD4 Treg cells and improved the prognosis of DN [100].

##### 4.3.2.2. Surface Functionalization

The exosome membrane can be chemically conjugated with targeting molecules (e.g., using click chemistry) to direct them to specific kidney cells, enhancing precision and reducing off‐target effects.

In summary, engineering exosomes—through either parent cell manipulation or direct post‐isolation modification—provides a powerful toolbox to optimize their targeting, cargo, and efficacy, offering a promising avenue for the next generation of DN therapeutics.

## 5. Challenges and Future Directions

### 5.1. Challenges Associated With Exosome Therapy

There are several limitations and challenges associated with exosome therapy, and some potential improvements could be explored deeply (Table 5).

**Table 5:** Challenges and potential improvements for exosomes therapy.

Challenges	Potential improvements
Heterogeneity and variability of exosomes	Further engineering exosomes
Limited understanding of mechanisms of action	Improving combination therapy
Delivery challenges	Optimization of manufacturing and isolation methods
Safety concerns	Preconditioning of donor cells
Regulatory challenges	Use of targeted delivery systems
Cost considerations	Evaluation of exosome dose and frequency

(1) Exosomes are heterogeneous in size, composition, and function, and their cargo can vary depending on the cell source and culture conditions. This can lead to variability in therapeutic efficacy and difficulty in standardizing the manufacturing process [101]. (2) Although exosomes have shown promising therapeutic effects in preclinical studies, their mechanisms of action are not fully understood. It is unclear which specific components of exosomes are responsible for their therapeutic effects and how they interact with recipient cells. (3) Exosomes are rapidly cleared from the circulation and have limited tissue distribution [102]. Therefore, effective delivery strategies need to be developed to target specific tissues and ensure sustained release of exosomes [103, 104]. (4) Although exosomes are generally considered to be safe and well‐olerated, there is a lack of long‐term safety data for exosome therapy. There is also a concern that exosomes could transfer pathogenic cargo, such as viruses or oncogenes, from the donor cells to the recipient cells [105, 106]. (5) The regulatory landscape for exosome therapy is currently uncertain, and there is a lack of clear guidelines for clinical development and manufacturing of exosome‐based products [107, 108]. (6) Exosome therapy is likely to be expensive, and the cost‐effectiveness of this approach needs to be carefully evaluated in comparison to other available therapies [109, 110]. Addressing these limitations and challenges will be critical for the successful development and clinical translation of exosome therapy.

### 5.2. Strategies for Improving the Therapeutic Efficacy of Exosomes

Several strategies can be employed to improve the therapeutic efficacy of exosomes: (1) Exosomes can be engineered to enhance their targeting, stability, and therapeutic payload. For instance, exosomes can be modified to express specific ligands that facilitate targeting to specific cells or tissues. In addition, exosomes can be engineered to express or carry specific therapeutic cargoes, such as drugs or RNA molecules, to enhance their therapeutic potential [111, 112]. (2) Exosome therapy can be combined with other therapies, such as drugs or cell‐based therapies, to enhance their therapeutic efficacy. Thus, exosomes can be combined with stem cell transplantation to enhance the regenerative potential of the transplanted cells [113]. (3) Standardization of exosome manufacturing and isolation methods can improve the quality and consistency of exosome preparations, which can enhance their therapeutic efficacy [114, 115]. (4) Preconditioning of donor cells, such as stem cells, can enhance the production and release of therapeutic exosomes. For example, preconditioning stem cells with hypoxia or growth factors can enhance the release of exosomes with regenerative properties [116, 117]. (5) Targeted delivery systems, such as nanoparticles or liposomes, can be used to enhance the tissue distribution and retention of exosomes, which can improve their therapeutic efficacy [118, 119]. (6) The optimal dose and frequency of exosome administration need to be determined to achieve maximal therapeutic efficacy while minimizing potential side effects [120, 121].

Stem cell‐derived exosomes contain a variety of molecules, including proteins, lipids, and RNA, that can have therapeutic effects. In recent years, there has been growing interest in the use of stem cell‐derived exosomes for the treatment of DN. Several preclinical studies have shown that stem cell‐derived exosomes can protect against kidney damage and improve kidney function in animal models of DN. However, more research is needed to determine the safety and efficacy of stem cell‐derived exosomes in humans.

In summary, exosomes can serve as carriers for loading small‐molecule drugs, biologics, and functional nucleic acids (such as miRNA/siRNA, CRISPR/Case9, etc.). They exhibit superior capabilities in penetrating biological barriers and optimizing biodistribution, enabling targeted delivery to specific cells and tissues by exosomal surface‐specific proteins. This enhances the targeting efficiency and stability of the therapeutic agents and even gene editing.

### 5.3. Future Directions for Research in the Field of Exosomes Therapy for DN

The use of exosome therapy for DN is a promising area of research, but there are several areas that need to be addressed in the future to further improve its efficacy and safety. First, there is a need for more preclinical and clinical studies to determine the optimal dose, route of administration, and long‐term safety and efficacy of exosome‐based therapies. These studies should also investigate the potential for combination therapy with other treatments to further enhance the therapeutic effect. Second, there is a need for a better understanding of the mechanisms by which exosomes exert their therapeutic effects. This knowledge can help optimize the design of exosome‐based therapies and identify new therapeutic targets. Third, there is a need for the development of standardized methods for the isolation, characterization, and quality control of exosomes. This will ensure the consistency and reproducibility of exosome‐based therapies across different research groups and clinical settings. Finally, there is a need for the development of new and innovative technologies for the large‐scale production of exosomes or engineered exosomes. This will enable the widespread use of exosome‐based therapies for the treatment of DN and other diseases.

Clearly, exosome therapy holds great promise for the treatment of DN, but there is still much to be learned and improved. Continued research in this field will pave the way for the development of more effective and safer exosome‐based therapies for the treatment of DN and other diseases.

## 6. Conclusion

This review highlights the therapeutic effect of exosomes as a promising approach for the treatment of DN. MSC‐Exos have shown therapeutic potential in preclinical and clinical studies and may offer advantages over other stem cell‐derived exosomes due to their immunomodulatory and regenerative properties. In addition, the review emphasizes the potential advantages and disadvantages of using exosomes derived from PSCs (iPSCs and ESCs) for DN therapy. Studies comparing exosomes derived from MSCs and PSCs have shown that MSC‐Exos have greater therapeutic potential for DN. Overall, the review underscores the need for further research to fully understand the differences between exosomes derived from different stem cell types and their potential for therapeutic applications in DN. Ultimately, optimizing effective exosome‐based therapies for DN could significantly improve patient outcomes and reduce the burden of this debilitating disease.

## Author Contributions

Yuanyuan Zhang and Anyong Yu conceived the thesis of this paper. Yuanyuan Zhang, Mo Li, and Tianjing Sun are major contributors in writing and reviewing the manuscript. Yuanyuan Zhang, Allen Gao, Andrew Huang, and Gagan Deep edited the manuscript. Anyong Yu finalized the manuscript.

## Funding

This study is supported by funding supports like Guizhou Province Science and Technology Plan Project (No. ZK2021‐General446), National Natural Science Foundation of China (No. 82060245 and 82260254); the National Institute of Allergy and Infectious Diseases, National Institutes of Health, under Contract No. R21 AI152832, R03 AI165170, and R21 EY035833 (PI: Yuanyuan Zhang).

## Disclosure

All authors read and approved the final manuscript.

## Conflicts of Interest

The authors declare no conflicts of interest.

## Data Availability

All data generated or analyzed during this study are available in this published article.
